# Progressive Phosphorylation Modulates the Self-Association of a Variably Modified Histone H3 Peptide

**DOI:** 10.3389/fmolb.2021.698182

**Published:** 2021-06-11

**Authors:** George V. Papamokos, George Tziatzos, Dimitrios G. Papageorgiou, Spyros Georgatos, Efthimios Kaxiras, Anastasia S. Politou

**Affiliations:** ^1^ Biomedical Division, The Institute of Molecular Biology and Biotechnology, FORTH-ITE, Ioannina, Greece; ^2^ Department of Physics and School of Engineering and Applied Sciences, Harvard University, Cambridge, MA, United States; ^3^ Laboratory of Biological Chemistry, Medical School, University of Ioannina, Ioannina, Greece; ^4^ Department of Materials Science and Engineering, University of Ioannina, Ioannina, Greece; ^5^ Laboratory of Biology, School of Medicine, University of Ioannina, Ioannina, Greece

**Keywords:** post-translational modification, protein phosphorylation, histone, peptide structure, chromatin, intrinsically disordered proteins, molecular dynamics, NMR

## Abstract

Protein phosphorylation is a key regulatory mechanism in eukaryotic cells. In the intrinsically disordered histone tails, phosphorylation is often a part of combinatorial post-translational modifications and an integral part of the “histone code” that regulates gene expression. Here, we study the association between two histone H3 tail peptides modified to different degrees, using fully atomistic molecular dynamics simulations. Assuming that the initial conformations are either α-helical or fully extended, we compare the propensity of the two peptides to associate with one another when both are unmodified, one modified and the other unmodified, or both modified. The simulations lead to the identification of distinct inter- and intramolecular interactions in the peptide dimer, highlighting a prominent role of a fine-tuned phosphorylation rheostat in peptide association. Progressive phosphorylation appears to modulate peptide charge, inducing strong and specific intermolecular interactions between the monomers, which do not result in the formation of amorphous or ordered aggregates, as documented by experimental evidence derived from Circular Dichroism and NMR spectroscopy. However, upon complete saturation of positive charges by phosphate groups, this effect is reversed: intramolecular interactions prevail and dimerization of zero-charge peptides is markedly reduced. These findings underscore the role of phosphorylation thresholds in the dynamics of intrinsically disordered proteins. Phosphorylation rheostats might account for the divergent effects of histone modifications on the modulation of chromatin structure.

## Introduction

Post-translational modifications (PTMs) of core and linker histones, either on their flexible parts or globular domains, are believed to be a part of a complex regulatory mechanism (Andrews et al., 2016a). The PTM machinery is currently understood on the basis of the “histone or epigenetic code” hypothesis (Strahl and Allis, 2000; Gardner et al., 2011; Soloway, 2016). As per this hypothesis, single histone modifications and their combinations alter the structure of chromatin and/or generate a binding platform for effector proteins, enabling gene activation or silencing (Hake and Allis, 2006). This hypothesis, however, is not the only interpretation of PTM functionality (Lee et al., 2010).

Protein phosphorylation is the most well-studied PTM and occurs in all eukaryotic and prokaryotic organisms. Protein phosphorylation maintains cellular functionality, regulates protein activity by serving as a digital molecular switch or rheostat, and reversibly determines cellular dynamics and plasticity. A survey of curated databases reporting experimentally verified PTMs across organisms reveals that phosphorylation is by far the most common PTM: more than one-third of all eukaryotic proteins undergo reversible phosphorylation (Khoury et al., 2011; Huang et al., 2016; Ullah et al., 2016).

The diverse chemistries of PTMs in histone proteins and the huge repertoire of possible combinations are indicative of a highly evolved and finely tuned machinery. Experimental evidence obtained with a broad range of technologies, in particular mass spectrometry, chromatin immunoprecipitation and *in vitro* assays, have proven that PTMs occur in patterns and operate in a combinatorial manner (Yuan et al., 2014; Huang et al., 2015; Savitsky et al., 2016; Soloway, 2016; Su and Denu, 2016). However, these techniques have limitations. For instance, analysis of histone peptides by mass spectrometry cannot easily distinguish between single PTMs located on the same molecule or in different molecules. Furthermore, chromatin immunoprecipitation could be affected by neighboring PTMs, which affect antibody binding to nucleosomes.

To overcome some of the limitations associated with current experimental techniques, particularly those concerning the identification of intra- and intermolecular interactions of combinatorial PTMs, we have employed molecular modeling and simulations, which are being increasingly integrated with biochemical and biophysical analyses (Samish et al., 2015). Focusing on the self-association of histone H3, we performed full atomistic molecular dynamics simulations for a variably modified part of the H3 flexible tail (aa 1–12). In addition, we studied the interactions between identically and variably modified oligopeptides corresponding to this sequence.

This approach was chosen for several reasons. First, it has been shown *in vivo* that in mitotic cells the H3 tail carries a set of modifications - phosphorylated T3, trimethylated K4, asymmetrically dimethylated R8–which represents a novel combinatorial pattern of histone PTMs, known as PMM (Markaki et al., 2009). This remarkably conserved pattern, established at the beginning of mitosis and completely erased at the end of cell division, appears to act as a determinant of chromatin folding. Second, the oligopeptide selected in this study is part of histone H3, which carries the most abundant and best characterized PTMs reported in the literature (Huang et al., 2015). This stretch of sequence (12 aa) allows *in silico* exploration of intra and intermolecular interactions for various combinations of experimentally identified PTMs. Finally, this oligopeptide is a representative example of a “linear peptide motif” with multiple PTM sites (Cortese et al., 2008; Tompa et al., 2014). Such motifs in intrinsically disordered proteins (IDPs) and intrinsically disordered regions (IDRs) are thought to provide short interaction interfaces, enabling exceptional functional plasticity and functional density to IDPs or IDRs that they are embedded in (Dyson and Wright, 2005; Oldfield and Dunker, 2014). The role of these motifs is critical in the formation of “fuzzy complexes” between IDPs and their targets, i.e., complexes in which the structural ambiguity of the IDP partner persists and the disordered state is maintained upon protein-protein interactions (Tompa and Fuxreiter, 2008; Sharma et al., 2015). The amino-terminal tail of H3 has all the features of a typical IDR (Hansen et al., 2006), and the oligopeptide under study is modifiable at multiple sites, making it an ideal model to study histone interactions.

Through simulations we could identify distinct inter and intramolecular interactions between differentially and identically modified peptides, characteristic of their association tendencies. Our findings highlight the dominant effect of progressive phosphorylation on peptide association, which is reversed when the negative charge of phosphate groups counterbalances the positive charge of peptide sequences. The present work is one of the first attempts to simulate the fine-tuning of a phosphorylation rheostat. These results should help elucidate the molecular mechanisms that guide the epigenetic machinery in large-scale systems.

## Methods

### Computational Methods

We performed Molecular Dynamics (MD) simulations for the following H3 (1–12 aa) oligopeptides with a C-terminal N-methyl amide capping group (tabulated and annotated in Table 1):

I. The unmodified peptide carrying no PTMs denoted here as P0M0 (ala): A_1_RTKQTARKSTG_12_-NMe, with +5 charge.
II. The peptide carrying the PMM pattern, denoted here as P1M2: A_1_RT_*phos*_K_*me3*_QTAR_*me2*_KSTG_12_-NMe, with total charge +3.
III. The peptide carrying the PMM pattern, with a capping acetyl group (ACE residue) replacing the Ala_1_ N-terminal amino acid and the remaining modifiable residues of the sequence modified as ACE-R_*me2*_T_*phos*_K_*me3*_QTAR_*me2*_K_*me3*_S_*phos*_TG-NMe. This peptide is fully modified and denoted as P2M4. The modifications were designed based on recent literature reports and the results of our simulations with P0M0 (ala) and P1M2. The ACE residue was introduced to neutralize the positive charge of the N-terminal residue and, along with the modifications of the additional Arg, Lys and Ser residues, resulted in a sequence with a total charge of 0.
IV. The unmodified peptide with an ACE residue replacing the Ala_1_ N-terminal aminoacid, denoted as P0M0 (ace): ACE-RTKQTARKSTG-NMe, with charge +4.

**TABLE 1:** Annotation, exact sequence and total charge of the H3 oligopeptides.

Annotation	Sequence	Charge
P0M0 (ala)	A_1_RTKQTARKSTG_12_-NMe	+5
P0M0 (ace)	CE-RTKQTARKSTG_12_-NMe	+4
P1M2	A_1_RT_*phos*_K_*me3*_QTAR_*me2*_KSTG_12_-NMe	+3
P2M4	ACE-R_*me2*_T_*phos*_K_*me3*_QTAR_*me2*_K_*me3*_S_*phos*_TG-NMe	0

Notes: ACE: N-terminal capping acetyl group; NMe: C-terminal N-methyl amide capping group; Kme_3_: trimethylated lysine residue; Rme2: asymmetrically dimethylated arginine residue; T_phos_: phosphorylated threonine residue; S_phos_: phosphorylated serine residue.

Dimers of the above H3 oligopeptides were generated as follows:

(1) both oligopeptides unmodified: P0M0 (ala)/P0M0 (ala),
(2) one peptide PMM-modified and one unmodified: P1M2/P0M0 (ala),
(3) both peptides PMM-modified: P1M2/P1M2,
(4) both peptides unmodified with ACE residue replacing the N-terminal Ala_1_: P0M0 (ace)/P0M0 (ace)
(5) one peptide fully modified and one unmodified with ACE residue replacing Ala_1_: P2M4/P0M0 (ace)
(6) both peptides fully modified with ACE residues replacing the N-terminal Ala_1_: P2M4/P2M4

The initial states adopted for all pairs were: 1) α-helical (Supplementary Figure S1) and 2) fully extended structures. Thus, 12 ensembles were generated and subsequently treated.

We employed the Amber parm99 force field (Cornell et al., 1995; Cheatham et al., 1999) as modified by Hornak et al. (ff99SB; Hornak et al., 2006). We used the parameters proposed by Craft and Legge (Craft and Legge, 2005) for phosphoserine and phosphothreonine and the partial charges previously derived by our group for methylated lysine and arginine residues (Papamokos et al., 2012). Each model system was placed at the center of a rectangular periodic simulation box with a 70 Å edge length. The box was then filled with about 9,000 water molecules modeled by the TIP3P approach (Jorgensen et al., 1983). Charged systems were rendered neutral by adding Cl^−^ counter-ions, as needed. To mimic physiological conditions, an additional concentration of 200 mM NaCl was imposed. For the computation of non-local (non-bonded) interactions in the potential energy function, we set the cut-off to 10 Å. For the long-range electrostatic interactions of charged particles in this periodic system, the Particle Mesh Ewald (PME) method was employed.

The energy minimization of the system proceeded in two steps: initially, we applied constrained minimization: the water molecules and the solute hydrogen atoms relaxed for 5 × 10^5^ conjugate gradient minimization steps with the heavy solute atoms kept frozen. Full minimization followed, with the RMS gradient criterion set to 10^–2^ kcal/mol/Å). The system was heated to 310 K for a time period of 240 ps, and a fixed temperature condition was applied with the Langevin thermostat (1 ns equilibration period). Equilibration for 1 ns under constant pressure at 1 atm–Langevin piston (Feller et al., 1995) followed, and the system was then left to propagate in time for 200 ns. The SHAKE constraints were applied, the time step was 2 fs, and the trajectory information was periodically recorded with a step of 2.5 ps.

The NAMD software package (Phillips et al., 2005) was used for the calculations described above, while trajectories were analyzed using AmberTools and in-house developed code. Pictorial representation of molecules and visualization of trajectories were performed using the VMD software (Humphrey et al., 1996). POTAMOS–Mass Spectrometry Calculator (Vlachopanos et al., 2014) was used to design the molecular simulations according to predefined charge distribution.

### PMM Peptide Synthesis

A peptide encompassing residues 1–19 of histone H3 carrying the PMM signature and a C-terminal Cys residue was synthesized using standard Fmoc-based solid-phase chemistry at Rockefeller University Proteomics Resource Center Core Facility.

### Circular Dichroism Spectroscopy

Circular dichroism (CD) spectra of the PMM synthetic peptide in the far-ultraviolet range were recorded on a Jasco J-810 spectropolarimeter interfaced with a Peltier element for temperature control and calibrated with (+)-10-camphorsulfonic acid. Measurements were performed either in 20 mM phosphate buffer, pH 6.9, 1 mM TCEP or in water/trifluoroethanol mixtures of various proportions, using peptide concentrations of 20 µM. Quartz 1 mm path-length cells were used (Hellma Cells Inc.). Spectra were recorded at 12°C, with 0.5 nm resolution and were baseline-corrected by subtracting the appropriate buffer spectrum at the same temperature. The combined absorbance of cell, sample and solvent was kept at less than one over the measured spectral range. CD intensity was expressed in mdeg.

### NMR Spectroscopy

NMR spectra of the PMM synthetic peptide at natural abundance were recorded on a 600 MHz Bruker Avance spectrometer equipped with a cryogenically cooled triple resonance 1H{13C/15N} probe. Measurements were performed in 20 mM phosphate buffer, pH 6.9 at final concentrations of 1.8 mM. 15N HSQC spectra were recorded at 12°C with 64 transients and 1,024 (1H) x 256 (15N) complex points and processed by zero-filling (ZF) to 4K and 512 points in the proton and nitrogen dimensions, respectively. iNMR 3.3.9 was used to process the data. Assignment of backbone amide NMR resonances was based on previous reports for the H3 tail (Liokatis et al., 2012).

## Results

**FIGURE 1:**
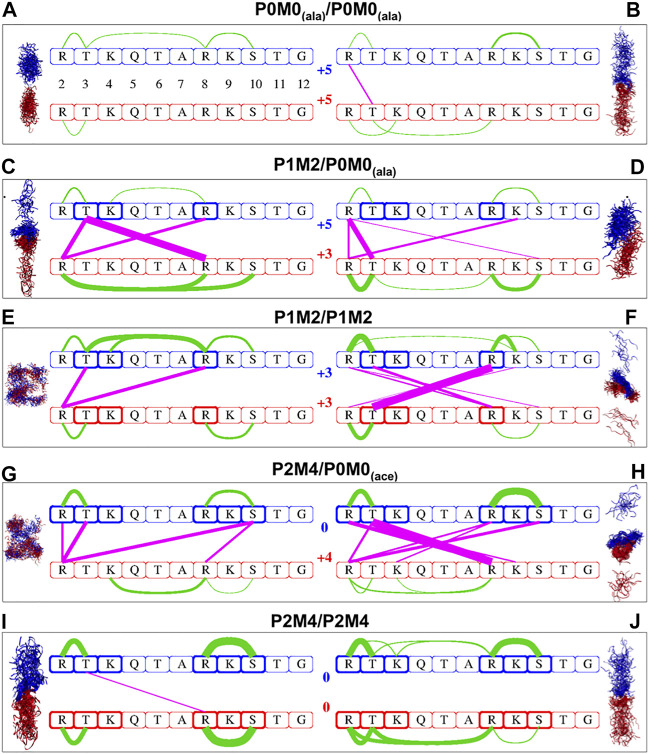
Intra- and intermolecular interactions in differentially modified H3 peptide pairs. Conformational sampling based on 80 frames per trajectory for each of the five pairs of differentially modified H3 peptides studied. Configurations derived from MD simulations for the originally α-helical conformations are shown on the left side, while those from initially extended conformations are shown on the right side. Red and blue ribbons represent the first and second peptide of each pair, respectively; water molecules are not included. The same color code is used in the linear schematic representation in the center with the H3 residues 2–12 in boxes. Green lines indicate intra-oligopeptide interactions and magenta lines inter-oligopeptide interactions; the thickness of the lines is proportional to the percentage of total simulation time during which the individual residues are in close contact, ranging from 10% of the time (thinnest lines) to 98% of the time (thickest lines). The residues in bold boxes are the ones that have been phosphorylated (T_3_, S_10_) or methylated (R_2_, K_4_, R_8_ K_9_).

Several intra- and intermolecular interactions between modifiable amino acids of the H3 peptides persisted during the MD simulations. The most persistent of those interactions and the percentage of total simulation time that each interaction was present for (using 10% as the lower threshold) are shown in Supplementary Table S1. A linear schematic representation of intra- and intermolecular interactions is shown in Figure 1, which also depicts conformational sampling based on 80 frames per trajectory for each of the five peptide pairs studied. These conformational ensembles are also shown in Supplementary Figure S2.

We determined the number of close contacts between the two peptides (N_cc_) and the change in solvent accessible surface area (ΔΑ) upon peptide association. Close contact was defined by a pair of heavy atoms at a distance <4 Å apart. Further, the excess solvent accessible surface area of the two separate monomers relatively to the dimer was calculated using the following equation:

$$\text{ΔA}\;=\;\langle {\text{A}}_{1}{+\text{A}}_{2}\;-\;{\text{A}}_{12}\rangle \text{,}$$

A_1_ and A_2_ refer to the solvent-accessible surface area of every single monomer and A_12_ represents the solvent accessible surface area of the dimer. The brackets <> indicate a time average over the entire trajectory. These data are included in Table 2.

**TABLE 2:** Close contacts and change in solvent-accessible surface area upon peptide interaction.

	—	—	α-helical		extended	
Peptide dimer	Phos. residues	Charge	N_cc_	ΔΑ(Å^2^)	Ncc	ΔΑ(Å^2^)
P0M0 (ala)/P0M0 (ala)	0	+5/+5	1.5	22	17.4	274
P1M2/P0M0 (ala)	1	+3/+5	42.1	495	26.1	332
P1M2/P1M2	2	+3/+3	29.9	373	52.0	632
P0M0 (ace)/P2M4	2	+4/0	33.7	405	58.6	762
P2M4/P2M4	4	0/0	23.0	364	19.7	253

Notes: Phos. residues: Number of phosphorylated residues; N_cc_: average number of close contacts between the two monomers in each pair, where a close contact is defined by a pair of heavy atoms being less than 4 Å apart; ΔA: excess solvent accessible surface area defined as ΔA = <A1 + A2–A12 > where A_1_, A_2_ refer to the solvent-accessible surface area of each peptidic chain considered alone and A_12_ refers to that of the pair. The brackets < > indicate a time average over the entire trajectory. For peptide pair annotation, see Table 1 and text.

As expected, the initially α-helical and fully extended structures were not observed at the end of the simulation.

### Extent of Association as a Function of the Charge and Number of Phosphorylation Sites

Analysis of simulation data revealed a general trend for more inter- and/or intramolecular peptide interactions with an increase in the number of PTMs. For example, the interactions between a fully modified and an unmodified peptide (P0M0_(ace)_/P2M4, Figures 1G,H) were much more extensive than those between two unmodified peptides (P0M0_(ala)_/P0M0_(ala)_, Figures 1A,B).

When no modifications were present, the intermolecular interactions were negligible: only one intermolecular interaction between R2 and T3, which was present for 19% of the total simulation time, was detected for the fully extended initial structures, while in the case of the initially α-helical peptides, no intermolecular interaction exceeded the threshold. Interestingly, although both chains were unmodified and positively charged, there was still a conformational space where they associated for almost 20% of the total simulation time (Supplementary Table S1). The results obtained for the other pair of unmodified sequences [P0M0_(ace)_/P0M0_(ace)_] were very similar and therefore are not discussed further.

Upon increase in the number of modifications, the sites and the extent of interactions increased too: for the PMM-modified/unmodified pair [P1M2/P0M0_(ala)_] the number of intermolecular interactions (*n*) for the initially α-helical conformation was *n* = 3, with one of these interactions being present for >94% of the total simulation time (Figure 1C); for the fully extended conformation, *n* was 4 (Figure 1D), with the most persistent interaction being present for 64% of the time (Supplementary Table S1). When both peptides were PMM-modified (P1M2/P1M2), *n* was two for the initially α-helical conformations and six for the fully extended ones (Figures 1E,F). The association was stronger and very persistent in the latter case (R8_*me2*_–T3_*phos*_, for 98% of the simulation time). For the fully modified/unmodified pair (P2M4/P0M0), the intermolecular interactions were even more extensive, i.e., *n* = 4 for the initially α-helical conformations and *n* = 7 for the fully extended ones (Figures 1G,H), with the latter engaged in full-time association, as evident via the persistence of the T3_*pho*_s–R8_*me2*_ interaction (Supplementary Table S1).

Surprisingly, in the case of the fully modified pair (P2M4/P2M4), the number of intermolecular interactions was comparable to that of unmodified peptides (Figures 1I,J): no interaction persisted for the fully extended initial structures, while for the initially α-helical peptides, the only remaining T3_*phos*_ *–*R8_*me2*_ interaction was absent for 85% of the simulation time (Supplementary Table S1). Two distinct intramolecular salt bridges between R8_*me2*_ and S10_*phos*_ and between R2_*me2*_ and T3_*phos*_ were present within each monomer.

From the number of close contacts between the peptides and the excess solvent accessible surface area (ΔΑ) of the two separate monomers relatively to the dimer (Table 2), it became evident that for the bimolecular system of two initially α-helical conformations, the most extensively associated dimer was formed between the PMM-modified and the unmodified peptide [P1M2/P0M0_(ala)_]. On the other hand, for the set of fully extended initial structures, the maximum intermolecular interaction was observed between the fully modified and the unmodified peptide [P2M4/P0M0_(ace)_], closely followed by the pair of PMM-modified peptides (P1M2/P1M2).

The major conclusions drawn from the data presented in Figure 1, Table 2 and Supplementary Table S1 can be summarized as follows:

(1) A single phosphorylation event was able to drive the two chains to maximum interaction.
(2) Progressive phosphorylation of the chains could fine-tune this association.
(3) The intermolecular interaction was minimized for the most extensively modified combination (P2M4/P2M4). This modification pattern resulted in monomers with zero charge.

In brief, it appears that the local saturation of positive charges by progressive phosphorylation has different outcomes on the association of corresponding peptides: up to a certain point, it can definitely induce strong intermolecular interactions between peptides, as long as they still carry an overall net charge. These intermolecular interactions are much less favored when extensive phosphorylation counterbalances the positive charge and peptides are no longer charged, although they still encompass a number of local dipoles.

### Most Persistent Interactions

In this study, the most persistent interactions (present for >90% of the total simulation time) existed chiefly between R-S_*phos*_ and between R-T_*phos*_ residues (Supplementary Table S1). Such interactions between histone PTMs and their reader proteins have been the focus of experimental and theoretical studies (Macdonald et al., 2005; Papamokos et al., 2012; Noh et al., 2015). With the exception of the fully modified dimer, we observed that the formation of a T3_*phos*_–R8 (methylated or unmodified) intermolecular salt bridge was followed by an almost full-time association (>94% of the simulation time) between the two peptides (see P1M2/P0M0 α-helical, P1M2/P1M2 extended and P2M4/P0M0 extended in Figure 1 and Supplementary Table S1). Interestingly, no interactions of the same type were detected between modified or unmodified K and phosphorylated S or T for the molecular systems studied herein. This finding was consistent with the fact that the side chain of R (pK_*a*_ > 12), a poor proton donor, cannot hydrolyze phosphorylated amino acids and can form substantially stronger salt bridges with phosphorylated side chains in comparison with Lys (Mandell et al., 2007).

### Multiplicity of Association Sites Depending on the Initially Adopted Conformations

We observed that the type of initial peptide conformations adopted for simulations had a considerable effect on the number of intermolecular interactions (Table 2, Supplementary Table S1 and Figure 1). Thus, identically modified monomers associated through a different network of intermolecular interactions depending on the structures initially selected (α-helical or fully extended). This effect could be attributed to the time limitations of a molecular dynamics study and, consequently, to the non-exhaustive conformational sampling, which may not allow all possible states of the molecular system to be revealed and surveyed by a single simulation. It could also result from the multiple modes of interaction likely to occur in such systems and is reminiscent of the “fuzziness” observed in IDP complexes, i.e., of a dynamic disorder resulting from the interconversion between multiple conformational states (Cortese et al., 2008; Tompa et al., 2014). The diverse functionality of this class of proteins is partially a manifestation of underdetermined molecular systems, where small perturbations can induce the formation of multiple conformational and functionally relevant states (Bhowmick et al., 2016).

Furthermore, it appears that intermolecular interactions are less favored in the case of α-helical structures than in the case of fully extended ones, at least in the timescale of the simulations performed. This is reasonable, considering that an α-helix is more compact, and its unfolding, i.e., the disruption of local intramolecular interactions and their replacement by intermolecular interactions, inevitably carries a high cost.

### Formation of an Aggregated or Collapsed PMM-Modified Peptide Is Excluded by CD and NMR Spectra

**FIGURE 2:**
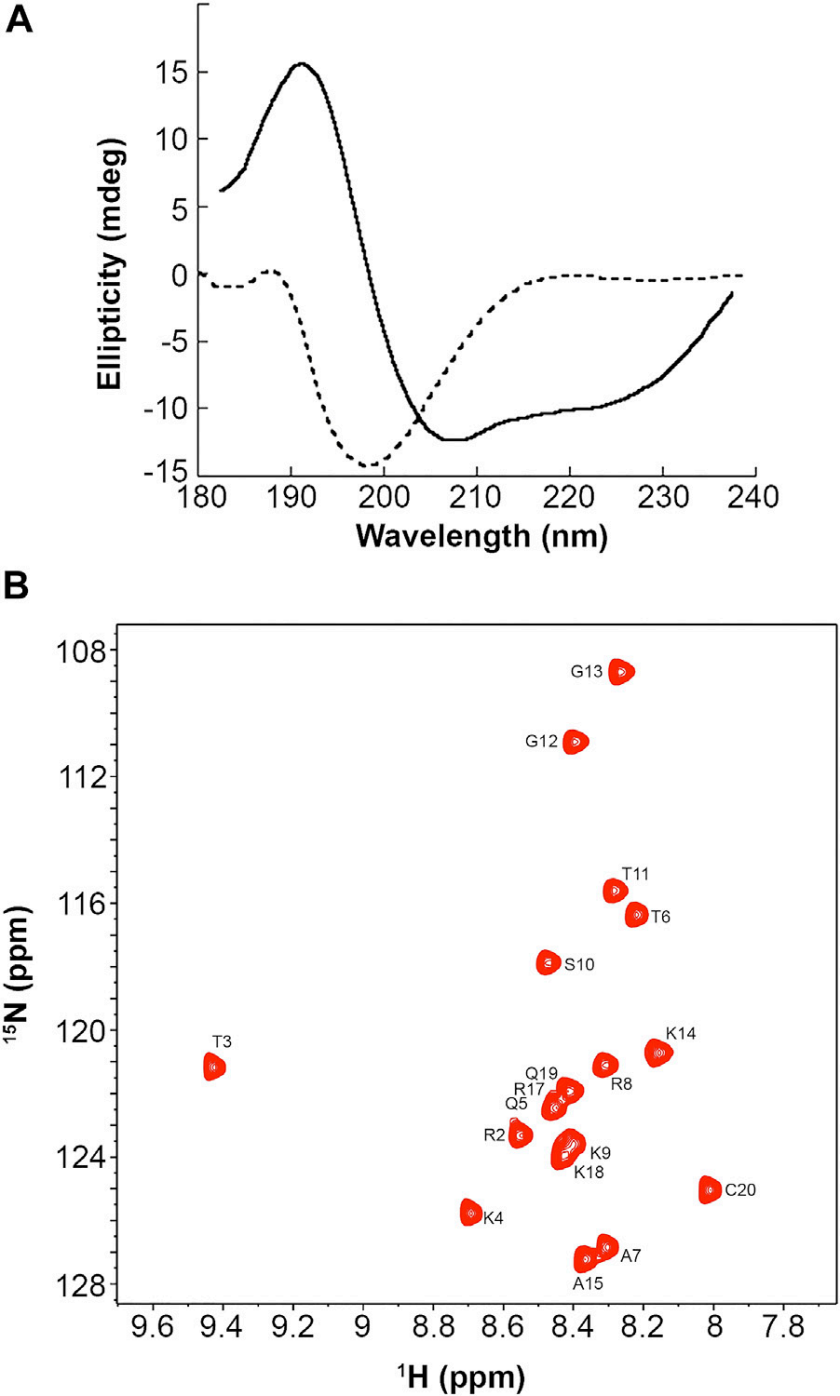
CD and NMR spectra of a synthetic PMM-modified H3 (1–19) peptide with sequence ART_phos_K_me3_QTAR_me2_KSTGGKAPRKQC. **(A)**: CD spectra of the peptide at 12°C in 20 mM phosphate buffer, 1 mM TCEP, pH = 6.9 (dashed line) and in 90% aqueous TFE (solid line). **(B)**: Natural-abundance ^15^N-HSQC spectrum of the peptide at 12°C in 20 mM phosphate buffer, 1 mM TCEP, pH = 6.9.

Our simulations showed that the PMM-modified histone tails associate through a set of specific and strong interactions. These interactions could support a mechanism of chromatin folding based on nucleosome association, provided that they occur in an orderly fashion and do not result in the formation of higher-order aggregates. To experimentally assess the physical state of these peptides we synthesized a H3 (1-19) peptide carrying the PMM-modifications and recorded CD spectra in water (Figure 2A, dashed line). These spectra showed that the peptide adopts no regular secondary structure. This is expected for peptides of this size, which, however, besides being disordered, quite often are also heavily aggregated. There was no such indication for PMM, as the CD pattern for aggregated states is markedly different than the one recorded here. No conclusions on the oligomeric state of the peptide could be drawn from CD spectra alone. However, we could safely exclude the formation of any amorphous or ordered aggregates and assume that the intra- and inter-molecular interactions present in PMM are specific and well defined.

It is also noteworthy that when the peptide was dissolved in aqueous trifluoroethanol (TFE), the CD pattern markedly changed, exhibiting minima at 222 and 208 nm and maximum at around 190 nm, which is typical of an α-helical secondary structure (Figure 2, solid line). This change was also observed in water and TFE, mixed in a ratio of 5:1, but was more pronounced in 90% aqueous TFE. Aqueous TFE is known to stabilize the native conformations of small peptides or protein fragments that exhibit no stable secondary structures in aqueous solutions and is widely considered as a probe of the inherent tendency of short sequences to adopt a secondary structure (Sonnichsen et al., 1992). Therefore, the PMM-modified peptide seems to preferentially adopt an α-helical secondary structure, in agreement with the theoretical prediction described above. This finding lends additional support to the hypothesis of non-aggregated state in water. Otherwise, the stabilizing effect of TFE would not have been so clear, as it is known that TFE induces or increases rather than alleviating the aggregation of disordered peptides (Anderson and Webb, 2012).

Natural abundance 15N-HSQC NMR spectra recorded for PMM under the same conditions corroborated the CD-derived results. As shown in Figure 2B, the dispersion, the linewidth and the intensity of the NMR signals were indicative of a peptide that is certainly not aggregated.

## Discussion

Our simulations led to the identification of distinct inter- and intramolecular interactions, characteristic of the association tendencies between post-translationally modified H3 peptide motifs. Among the different types of PTMs, phosphorylation seems to play a dominant role in the association between differentially or identically modified H3 peptides. The charge of peptides is modulated by the extent of phosphorylation, which supports strong intra- and intermolecular ionic interactions between phosphorylated S/T and R residues. Similar results have been previously reported for the role of R–S_*phos*_ salt bridges in CLASP2/EB1 binding (Kumar et al., 2012). Our simulation data highlight another theme that, to the best of our knowledge, has not been described before in the context of IDPs: while an increasing number of intra- or intermolecular salt bridges are formed upon progressive phosphorylation, a crucial plateau is reached when the accumulated negative charge counterbalances the positive charge of the peptide motif. The phosphate groups at the zero charge state are preferentially engaged in intramolecular rather than intermolecular interactions and the association of the two peptide chains is considerably reduced.

Simulation results might represent a real effect or be a consequence of inadequate sampling of the conformational and interaction space of the tested peptides during the timescale of the simulations. To address this issue, one could choose peptides of different lengths and modifications, run the simulation for a longer duration, and use enhanced sampling techniques or various force fields. However, in this particulat case, this might not be necessary, as our conclusions are strongly supported by theoretical and experimental data from several independent sources: modified histone tails and IDPs/IDRs are typical polyampholytes; it has been long known that the shift of a polyampholyte to a zero-charge chain turns it into a collapsed globule due to electrostatic interactions (Higgs and Joanny, 1991; Müller-Späth et al., 2010). In other words, neutralization of the total charge markedly affects the physical state of polyampholytes, as also observed in the case of the histone peptide in this study. Furthermore, PTM and, more specifically, progressive phosphorylation of proteins can result in the shift of their isoelectric point by several pH units, an effect more pronounced for proteins with isoelectric point >6.4 (Zhu et al., 2005). The adjustment of protein charge has served as the basis of several methods used for protein separation and characterization. To summarize, progressive phosphorylation and zero-point charge are the key mechanisms supported by theory and exploited in biochemistry.

Several groups have studied the effect of charge distribution on the conformations of intrinsically disordered proteins. Pappu and coworkers reported a theoretical framework to predict the dimensions and internal structure of polyampholytic IDPs based on a fraction of charged residues and sequence-specific distributions of oppositely charged residues (Das and Pappu, 2005; Mao et al., 2010). They also found that global conformational properties of an 81-residue IDR from the *Saccharomyces cerevisiae* transcription factor Ash1, prior to and upon multisite phosphorylation at 10 distinct sites, were indistinguishable (Martin et al., 2016). This finding was attributed to “linear sequence patterning of Pro and charged residues vis-à-vis all other residues.” Our findings provide a strong indication that the functionality of IDPs, in the framework of multisite phosphorylation, should be studied beyond single-molecule approaches (Zhang et al., 2012) and that the state of zero charge is critical for a phosphorylation rheostat-like mechanism.

The importance of charge-mediated interactions involving binding interactions between IDPs was also recently highlighted by the experimental detection and molecular simulations of a high-affinity and highly dynamic complex formed by two oppositely charged IDPs-prothymosin and histone H1. This association stems from the fact that these proteins carry a large charge opposite to each other, and in contrast to the current model for molecular recognition, the formation of a well-defined structure or folded domains by the partners, the involvement of specific binding sites on the protein surface, or the potential for specific interactions between individual residues is not needed (Borgia et al., 2018).

Focusing on the charge induced upon the phosphorylation of a given IDPIDR, we can schematically represent the process using this simple equation:

$$\frac{\text{IDP}}{\text{IDR}}\;+\;{x\text{.PO}}_{3}{\;}^{-2}\;\to \;\frac{\text{IDP}\;{\left({\text{PO}}_{3}{\;}^{-2}\right)}_{x}}{{\left({\text{IDRPO}}_{3}{\;}^{-2}\right)}_{x}}.$$

Considering the chemical biology of progressive phosphorylation, how and to what extent the different values of *x* can modulate the regulatory role of IDP/IDR phosphorylation? This question has been experimentally addressed in several systems (Wright and Dyson, 2015). For example, it has been suggested that phosphorylation can induce aggregation from the native to the amyloid state (Kardos et al., 2015). In contrast, progressive phosphorylation of the FRQ protein has been shown to support the time-delayed regulative mechanism of the circadian clock in *Neurospora*. Opposing effects of spatially and temporally regulated phosphorylation on overt circadian rhythms and FRQ stability were directly demonstrated in this case (Tang et al., 2009; Querfurth et al., 2011). Along the same lines, accumulated experimental evidence suggests that progressive phosphorylation of IDPs serves as a mechanism for the fine-tuning of their cellular concentration, which is normally very low in multicellular organisms and its aberrant increase is lethally toxic for cells (Gsponer et al., 2008). This is an essential function, as the fidelity of IDP-mediated cellular signaling relies on the precise regulation of IDP abundance. Consequently, the implications of the chemical biology in the above equation are not limited to *in vitro* systems; they seem to support a crucial regulatory mechanism in the cellular context as well.

Despite extensive studies, the molecular mechanisms that govern the regulation of essential biological processes *via* phosphorylation have not been identified in their entirety. Until recently, it was believed that phosphorylation events mimic molecular switches that activate or inactivate protein functions either directly or by allostery. However, it appears that phosphorylation thresholds are of critical importance (Borg et al., 2007). More recently, it was suggested and experimentally documented that multisite phosphorylation also operates as a “rheostat” (Pufall et al., 2005; Garske et al., 2010; Bah et al., 2015; Humphrey et al., 2015; Andrews et al., 2016b; Vlastaridis et al., 2017). According to this model, several phosphorylation sites in a protein region, irrespective of their precise position, collectively contribute to the modification of proteins and form clusters, which operate as the actual functional units. Further, multiple phosphorylation sites in the disordered region of the transcription activator Ets-1 were reported to act additively to produce graded DNA binding affinity, indicating that gradual phosphorylation resembles an electric “rheostat” that is able to program transcription at the level of DNA binding (Miles et al., 2005). In addition, a series of experimental and theoretical studies on the interaction between the intrinsically disordered cyclin-dependent kinase inhibitor Sic1 and Cdc4 with variable phosphorylation levels have highlighted the critical role of cumulative electrostatic interactions in the phosphorylation-dependent ultrasensitive binding of intrinsically disordered ligands (Nash et al., 2001; Liu et al., 2014).

In the present study, we attempted to simulate the tuning of a phosphorylation rheostat at the molecular level in a model system, which, despite its small size, is still representative, biologically relevant, and closely related to chromatin organization. Our findings support the significant role of phosphorylation thresholds and charge modulation in IDP/IDR functionality and might, in part, account for the divergent effects of histone phosphorylation on chromatin structure modulation.

The role of histone phosphorylation in multiple cellular processes associated with chromatin remodeling and gene expression is dual: the same phosphorylation event can be involved both in chromatin condensation during mitosis and in chromatin relaxation and transcriptional activation (Cheung et al., 2000; Rossetto et al., 2012). Thus, the ultimate outcomes of histone phosphorylation seem to be dictated by an intricate interplay among several factors, facilitating a sophisticated control of chromatin remodeling. Such factors could undoubtedly be the cellular context, the local combinatorial PTM patterns that recruit distinct effector proteins, the cross-talk with other modifications, and phosphorylation rheostats of the sort described in this study for the H3 peptide motif.

The importance of charge modulation in proteins by progressive phosphorylation can only be explored by combining experimental work and computer simulations scaled on molecular systems of variable size (from peptide motifs to entire IDPs, e.g., histone tail modifiable motifs, full-length histones, and even nucleosomes). Such studies should systematically investigate the role of the zero-charge state and the transition from positively to less positively or negatively charged species, as in the case of the FRQ protein or Sic1 N-terminal region (Querfurth et al., 2011; Liu et al., 2014). Moreover, the kinetics of the association should be investigated (Zhou et al., 2012). Along these lines, a crucial issue is a comprehensive implementation of state-of-the-art integrative structural biology and biophysics and molecular simulation methodology, in the form of a “computational beamline,” which will enable the identification of key features of the “dark proteome”, i.e., of protein regions that cannot be mapped onto any existing structures in the Protein Data Bank (Bhowmick et al., 2016).

## Conclusion

We studied the association between phosphorylated and variably modified histone H3 tail peptides based on a novel combinatorial pattern identified *in vivo* using fully atomistic molecular dynamics simulations. Novel information was obtained on intra- and intermolecular interactions between histone H3 tails and on the crucial role of a phosphorylation rheostat in their association. Progressive phosphorylation modulates the peptide charge and induces strong intermolecular interactions between histone tails. However, upon complete saturation of positive charges by phosphate moieties, this effect was reversed, i.e., intramolecular interactions prevailed and dimerization of zero-charge peptides was markedly reduced. These findings highlight the significant role of charge modulation in progressive phosphorylation-based regulatory mechanisms and are corroborated by theoretical and experimental results reported in the literature. The comprehensive elucidation of these mechanisms demands further experimental studies and systematic simulations of several IDPs, including histones, in hierarchical scales. It is, nevertheless, already evident from several studies, including ours, that phosphorylation can operate as a molecular rheostat and that the zero-charge state might be one of the rheostat states that should be further investigated for their regulatory role.

## Data Availability

The raw data supporting the conclusion of this article will be made available by the authors, without undue reservation.
