# Supplementary material for: Progressive Phosphorylation Modulates the Self-Association of a Variably Modified Histone H3 Peptide
Source: Front Mol Biosci. 2021 Jun 11;8:698182. doi: 10.3389/fmolb.2021.698182 (PMC8226166; doi:10.3389/fmolb.2021.698182)
Supplement: Supplementary file 1 [file DataSheet1.PDF]

Supplementary material

Progressive Phosphorylation Modulates the Self-Association of a Variably Modified Histone H3 Peptide

George V. Papamokos[1,2,3,*], George Tziatzos[4], Dimitrios G. Papageorgiou[4], Spyros Georgatos[1,5], Efthimios Kaxiras[2], and Anastasia S. Politou[1,3,*]

[1]The Institute of Molecular Biology and Biotechnology, Biomedical Division, FORTH-ITE, GR-45110 Ioannina, Greece

[2]Department of Physics and School of Engineering and Applied Sciences, Harvard University, 02138 Cambridge, MA USA

[3]Laboratory of Biological Chemistry, Medical School, University of Ioannina, GR-45110 Ioannina, Greece

[4]Department of Materials Science and Engineering, University of Ioannina, GR-45110 Ioannina, Greece

[5]Laboratory of Biology, University of Ioannina, School of Medicine, GR-45110 Ioannina, Greece

***Correspondence:**

George V. Papamokos

gpapamokos@seas.harvard.edu

Anastasia S. Politou

apolitou@cc.uoi.gr

Keywords: post-translational modification, protein phosphorylation, histone, peptide structure, chromatin, intrinsically disordered proteins, NMR, molecular dynamics

Running title: Phosphorylation and histone association

Table S1 | Persistent intra- and intermolecular interactions in peptide dimers.

**α-helical**			**Fully extended**		
**Time %**	**Pair**	**Peptide**	**Time %**	**Pair**	**Peptide**
**1:unmodified (charge +5) / 2:unmodified (charge +5)**					
**P0M0$_{(ala)}$/P0M0$_{(ala)}$**					
16	R2-T3	1	26	R8-S10	1
13	R8-S10	1	19	R2-T3	X
12	R2-T3	2	14	T3-R8	2
12	T3-R8	1	12	R2-T3	1
			10	R2-K4	2
**1:PMM-modified (charge +3) / 2: unmodified (charge +5)**					
**P1M2/P0M0$_{(ala)}$**					
94	$T3_{phos}$ -R8	X	65	R2-T3	2
55	R2-S10	2	64	R2-T3	X
55	R2-R8	2	50	R8-S10	2
54	$T3_{phos}$ -R2	X	28	R2-R2	X
43	R2-R8	X	22	R2-K9	X
21	R2-$T3_{phos}$	1	16	R2-$T3_{phos}$	1
11	$K4_{me3}$-$R8_{me2}$	1	14	T3-R8	2
			13	$R8_{me2}$-S10	1
			11	R2-S10	X
**1:PMM-modified (charge +3) / 2:PMM-modified (charge +3)**					
**P1M2/P1M2**					
62	$T3_{phos}$ -$R8_{me2}$	1	98	$R8_{me2}$-$T3_{phos}$	X
45	$T3_{phos}$-R2	X	77	R2-$T3_{phos}$	1
44	$R8_{me2}$-R2	X	52	R2-$T3_{phos}$	2
38	R2-$T3_{phos}$	2	51	$R8_{me2}$-K9	1
34	$K4_{me3}$-$R8_{me2}$	1	37	$T3_{phos}$-$R8_{me2}$	X
29	R2-$T3_{phos}$	1	22	R2-$R8_{me2}$	X
28	$R8_{me2}$-S10	1	21	$R8_{me2}$-R2	X
16	$R8_{me2}$-S10	2	17	$R8_{me2}$-S10	2
			16	$R8_{me2}$-S10	1
			12	K9-R2	X
			10	R2-K9	1
			10	R2-S10	X
**1:fully modified (charge 0) / 2:unmodified (charge +4)**					
**P2M4/P0M0$_{(ace)}$**					
52	R_{me2}-$T3_{phos}$	1	97	$T3_{phos}$ -R8	X
50	$T3_{phos}$-R2	X	91	$R8_{me2}$-$S10_{phos}$	1
42	$S10_{phos}$-R2	X	59	$R2_{me2}$-$T3_{phos}$	1
41	$R8_{me2}$-$S10_{phos}$	1	49	$R2_{me2}$-R8	X
36	$R2_{me2}$-R2	X	43	$S10_{phos}$-R2	X
35	K4-R8	2	29	$T3_{phos}$-R2	X
28	$S10_{phos}$-R8	X	24	$R8_{me2}$-R2	X
10	R8-S10	2	23	R2-R8	2
			21	$R8_{me2}$-K4	X
			20	$R2_{me2}$-K9	X
			15	R2-T3	2
			11	K4-R8	2
**1:fully modified (charge 0) / 2:fully modified (charge 0)**					
**P2M4/P2M4**					
99	$R8_{me2}$-$S10_{phos}$	1	81	$R2_{me2}$-$T3_{phos}$	1
97	$R8_{me2}$-$S10_{phos}$	2	79	$R8_{me2}$-$S10_{phos}$	1
69	$R2_{me2}$-$T3_{phos}$	1	59	$R2_{me2}$-$T3_{phos}$	2
56	$R2_{me2}$-$T3_{phos}$	2	51	$T3_{phos}$ -$R8_{me2}$	2
15	$T3_{phos}$-$R8_{me2}$	X	48	$R2_{me2}$-$R8_{me2}$	2
			14	$T3_{phos}$ -$R8_{me2}$	1
			13	$R8_{me2}$-$S10_{phos}$	2
			13	$R2_{me2}$-$K4_{me2}$	1
			10	$K4_{me2}$-$R8_{me2}$	1

Interactions in the peptide dimers persistent for more than 10% of the total simulation time have been included. Intramolecular interactions are denoted with numbers (1 for those in the first peptide or 2 for those in the second peptide) and intermolecular interactions with X.

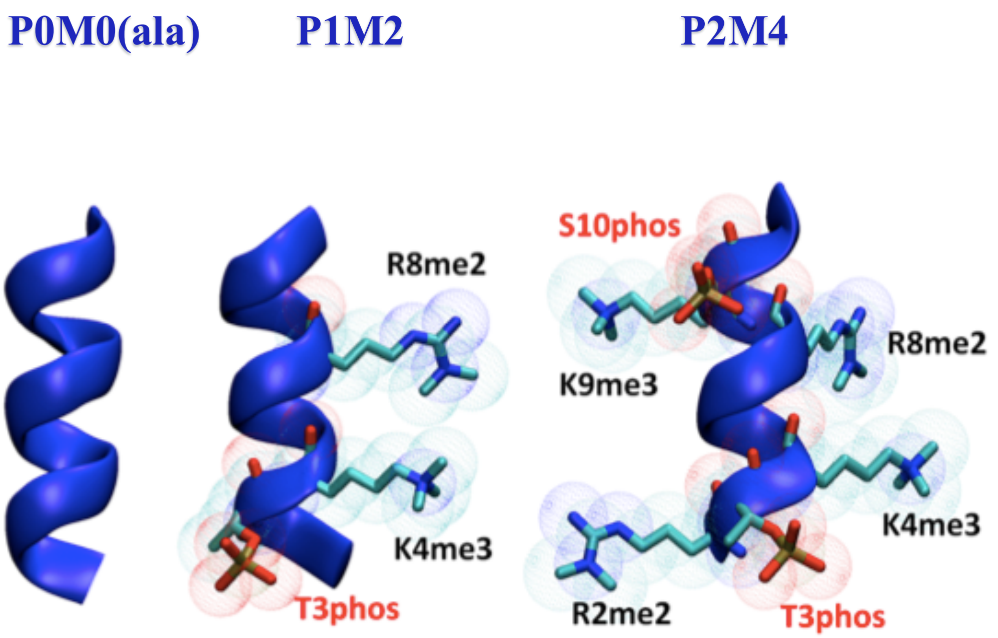

FIGURE S1 | Initial α-helical structures of the unmodified (P0M0(ala)), PMM-modified (P1M2) and fully modified (P2M4) peptide.

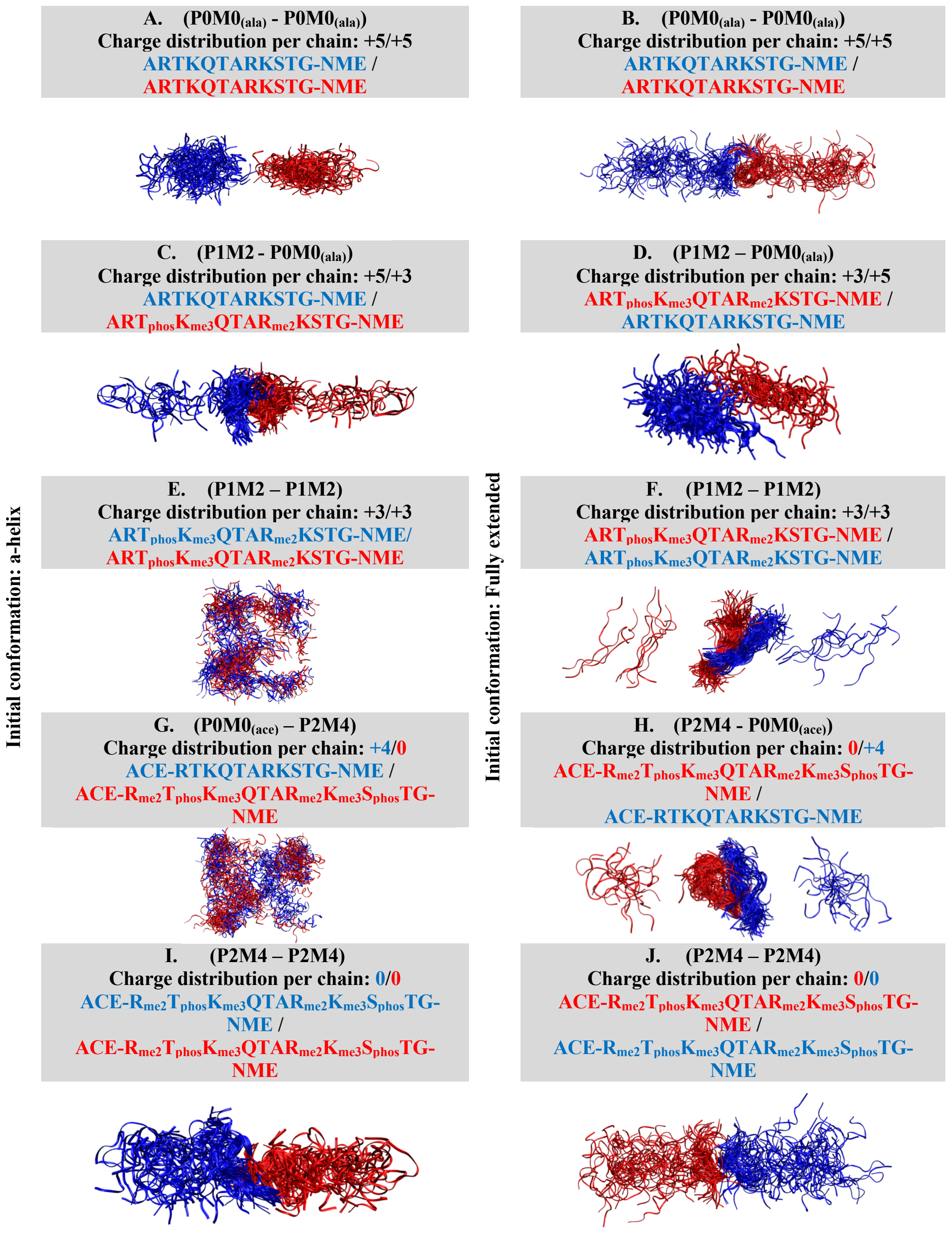

FIGURE S2 | Conformational sampling resulting in 80 frames per trajectory for each bimolecular system under study. Red and blue ribbons represent the two respective oligopeptides. Water molecules are not included. The results of simulations for peptides in an initially alpha-helical conformation are shown in panels A, C, E, G, I and those for peptides in an initially fully extended conformation in panels B, D, F, H, J. The exact sequence, the annotation (as in Table 2) and the charge of each peptide studied are included in the header of each panel .
